# Psilocybin for dementia prevention? The potential role of psilocybin to alter mechanisms associated with major depression and neurodegenerative diseases

**DOI:** 10.1016/j.pharmthera.2024.108641

**Published:** 2024-06

**Authors:** Zarah R. Haniff, Mariia Bocharova, Tim Mantingh, James J. Rucker, Latha Velayudhan, David M. Taylor, Allan H. Young, Dag Aarsland, Anthony C. Vernon, Sandrine Thuret

**Affiliations:** a Department of Basic and Clinical Neuroscience, Institute of Psychiatry, Psychology and Neuroscience, King's College London, United Kingdom; b MRC Centre for Neurodevelopmental Disorders, King's College London, United Kingdom; c Department of Old Age Psychiatry, Division of Academic Psychiatry, Institute of Psychiatry, Psychology and Neuroscience, King's College London, United Kingdom; d Department of Psychological Medicine, Institute of Psychiatry, Psychology and Neuroscience, King's College London, United Kingdom; e South London and Maudsley NHS Foundation Trust, Maudsley Hospital, Denmark Hill, London, United Kingdom; f South London and Maudsley NHS Foundation Trust, Bethlem Royal Hospital, Monks Orchard Road, Beckenham, Kent, United Kingdom; g Wolfson Centre for Age Related Diseases, Division of Neuroscience of the Institute of Psychiatry, Psychology and Neuroscience, King's College London, United Kingdom; h Stavanger University Hospital, Stavanger, Norway

**Keywords:** Dementia, Depression, Hippocampal neurogenesis, Microglia, Psilocybin

## Abstract

Major depression is an established risk factor for subsequent dementia, and depression in late life may also represent a prodromal state of dementia. Considering current challenges in the clinical development of disease modifying therapies for dementia, the focus of research is shifting towards prevention and modification of risk factors to alter the neurodegenerative disease trajectory. Understanding mechanistic commonalities underlying affective symptoms and cognitive decline may reveal biomarkers to aid early identification of those at risk of progressing to dementia during the preclinical phase of disease, thus allowing for timely intervention. Adult hippocampal neurogenesis (AHN) is a phenomenon that describes the birth of new neurons in the dentate gyrus throughout life and it is associated with spatial learning, memory and mood regulation. Microglia are innate immune system macrophages in the central nervous system that carefully regulate AHN via multiple mechanisms. Disruption in AHN is associated with both dementia and major depression and microgliosis is a hallmark of several neurodegenerative diseases.

Emerging evidence suggests that psychedelics promote neuroplasticity, including neurogenesis, and may also be immunomodulatory. In this context, psilocybin, a serotonergic agonist with rapid-acting antidepressant properties has the potential to ameliorate intersecting pathophysiological processes relevant for both major depression and neurodegenerative diseases. In this narrative review, we focus on the evidence base for the effects of psilocybin on adult hippocampal neurogenesis and microglial form and function; which may suggest that psilocybin has the potential to modulate multiple mechanisms of action, and may have implications in altering the progression from major depression to dementia in those at risk.

## 1 Introduction

Globally, the World Health Organisation (WHO) reports that over 55 million people are currently diagnosed with dementia, while an estimated 280 million people suffer from major depression; where the prevalence of depression is higher among adults aged 60 years and older (World Health Organisation, 2023, World Health Organisation, 2023). There is a complex inter-relationship between major depression and dementia. Specifically, a diagnosis of major depressive disorder (MDD) is associated with a 1.5-fold increased likelihood of a dementia diagnosis (Brzezińska et al., 2020; Cherbuin, Kim, & Anstey, 2015; Diniz, Butters, Albert, Dew, & Reynolds, 2013; Fernández Fernández, Martín, & Antón, 2023; Gracia-García et al., 2015; Liu et al., 2023; Livingston et al., 2020; Ownby et al., 2006; Yang et al., 2023). It follows that with a rising prevalence of both conditions more people may be at increased risk of developing dementia if their major depression is not effectively managed (Elser et al., 2023). Therefore, the current lack of safe, disease-modifying therapies that prevent, halt or reverse dementia signifies a major unmet medical need (Alves, Kallinowski, & Ayton, 2023; Andrews et al., 2019; van Dyck et al., 2022).

We need to understand common mechanisms underlying risk and resilience in the context of major depression and the development of dementia. Understanding how MDD and neurodegenerative diseases leading to dementia are connected mechanistically may aid identification of those at risk of developing dementia during the preclinical phase to allow for earlier intervention. One mechanism of interest is adult hippocampal neurogenesis (AHN), a phenomenon characterised by the generation of new neurons in the hippocampus, which is suggested to be essential for maintaining and regulating processes associated with learning, memory and mood throughout life. Important modulators of AHN are microglia; macrophages in the central nervous system (CNS), derived from erythromyeloid progenitor cells in the yolk sac that colonise the brain during neurodevelopment (Pérez-Rodríguez, Blanco-Luquin, & Mendioroz, 2021). Pertinently, disturbances in AHN are associated with both clinical depression and neurodegenerative diseases (Arber et al., 2019; Arber et al., 2021; Boldrini et al., 2013; Boldrini et al., 2019; Moreno-Jiménez et al., 2019; Salta et al., 2023; Tartt, Mariani, Hen, Mann, & Boldrini, 2022; Terreros-Roncal et al., 2021; Tobin et al., 2019; Zhou et al., 2022). Shifts towards disease-associated microglial functional states are associated with neurodegeneration and subsequent cognitive decline (Al-Onaizi, Al-Khalifah, Qasem, & Elali, 2020; Appel et al., 2018; Santos, Beckman, & Ferreira, 2016; Xu, He, & Bai, 2016). Therefore, compounds that modulate the neurobiological mechanisms overlapping both conditions may be beneficial for treating depressive symptoms and preserving cognitive function by altering the underlying pathology and subsequent trajectory of neurodegeneration.

Interest in psychedelics, such as psilocybin (4-phosphoryloxy-*N*,*N*-dimethyltryptamine), for the treatment of mental health conditions has increased significantly in recent years (Butlen-Ducuing et al., 2023; da Costa, Oesterle, Rummans, Richelson, & Gold, 2022). In various clinical trial designs, psilocybin-assisted psychotherapy has demonstrated rapid amelioration of depressive symptoms, in some patients with MDD and treatment-resistant depression (TRD) (Davis et al., 2021; Goodwin et al., 2023; Raison et al., 2023; von Rotz et al., 2022). In some cases, prolonged antidepressant effects have persisted for up to 12 weeks (Carhart-Harris et al., 2021; Carhart-Harris et al., 2018; Gukasyan et al., 2022). Further studies investigating the benefit of psilocybin specifically in TRD are underway (Rucker et al., 2021).

Emerging evidence suggests that psilocin, the active metabolite of psilocybin, may be a “psychoplastogen”, a term that suggests it mediates its effects via the promotion of neural plasticity (de Vos, Mason, & Kuypers, 2021; Moliner et al., 2023; Olson, 2018; Vargas et al., 2023a; Vargas, Meyer, Avanes, Rus, & Olson, 2021). Psilocin may also have anti-inflammatory activity (Szabo, 2015). Mechanisms associated with AHN and microglial activity produce and regulate the number of newly generated neurons in the hippocampus; the resultant influence on synaptic architecture constitutes a form of neuroplasticity.

This narrative review aims to elucidate the relationship between AHN and microglial activity, identifying them as mechanisms underpinning major depression and the associated increase in dementia incidence. It examines the dynamic interplay between AHN and microglia in regulating neurogenesis and shaping hippocampal synaptic architecture, a key aspect of neuroplasticity. We will consider the potential of psychedelic compounds, namely psilocybin, to promote neuroplasticity, potentially affecting these processes through direct or indirect interactions with hippocampal stem cells and microglia in the dentate gyrus. Since depression is a risk factor for developing dementia, the neuroplastic and immunomodulatory potential of psilocybin to moderate cellular mechanisms associated with AHN and microglia, at the interface of depression and dementia, may influence neurodegenerative pathology and cognitive function, and may be relevant for reducing dementia risk.

## 2 The relationship between major depression and dementia

### 2.1 Major depression is associated with increased dementia risk

Dementia is a chronic syndrome encompassing numerous neurodegenerative diseases that lead to irreversible, progressive neuronal loss resulting in significant cognitive impairment. This disruption pervades multiple domains affecting memory, language, visuospatial and motor function, and is often associated with behavioural and neuropsychiatric symptoms such as major depression, apathy, anxiety and agitation (Elahi & Miller, 2017; Laganà et al., 2022). Currently, the majority of licensed pharmacological treatments available for dementia patients, such as acetylcholinesterase inhibitors (donepezil, rivastigmine and galantamine) and the NMDA receptor antagonist (memantine) manage cognitive symptoms only (Knight, Khondoker, Magill, Stewart, & Landau, 2018) but do not prevent neurodegeneration and subsequent cognitive decline.

The most prevalent types of dementia are due to Alzheimer's disease (AD), Dementia with Lewy bodies (DLB), Parkinson's disease (PD), and frontotemporal dementia (FTD), and share common disease mechanisms despite being attributed to different pathological proteins (Erkkinen, Kim, & Geschwind, 2018). Therapies that target the underlying pathology associated with AD, the most researched and commonest form of dementia, are starting to emerge on the market (Dunn, Stein, & Cavazzoni, 2021; Sims et al., 2023). However, there are concerns regarding safety and efficacy of such monoclonal antibody therapies designed to remove β-amyloid (Aβ-42) aggregates, a neuropathological hallmark of AD (Salloway et al., 2022). The challenges associated with clinical development of DMTs for dementia are complex and multifactorial. Dementia presents insidiously, with varying and often mixed pathophysiologies, aetiologies and trajectories; which have been considerably difficult to recapitulate in preclinical models (McKean, Handley, & Snell, 2021). Variation in clinical presentation, compensatory mechanisms, probable and delayed diagnoses may all contribute to clinical trial failure due to significant neuronal atrophy and inappropriate treatment assignment at the point of intervention (Yiannopoulou, Anastasiou, Zachariou, & Pelidou, 2019). Therefore, focus is shifting towards early identification and prevention of dementia.

Major depressive disorder (MDD) is associated with a heterogeneous presentation of persistent low mood with anhedonia, apathy, and increased risk for suicide. Sleep disturbances and weight changes also occur and all of the above significantly impact on daily living. Notably, clinical depression is also known to manifest with cognitive symptoms affecting several domains including memory, executive function, processing speed and attention (Carvalho et al., 2014; Nuño, Gómez-Benito, Carmona, & Pino, 2021; Pan et al., 2019; Sampath, Sathyanesan, & Newton, 2017; Vancappel et al., 2021; Zuckerman et al., 2018). These cognitive domains may also be impaired in dementia (Kirova, Bays, & Lagalwar, 2015). Cognitive impairment may affect up to 94% of those during a depressive episode (Conradi, Ormel, & De Jonge, 2011). Furthermore, the prevalence of depression is estimated to be 32% (95% CI, 27-37) in those with mild cognitive impairment (MCI), a probability state associated with increased risk of progression to dementia (Roberts et al., 2014). Taken together, this indicates mood and cognitive function are inherently interconnected, and points to the potential co-occurrence of common underlying mechanisms between major depression and dementia that may possibly be related to cognitive dysfunction commonly co-occurring in MDD (Lam, Kennedy, McIntyre, & Khullar, 2014; Perini et al., 2019).

Late-life depression in particular - a term referring to a depressive episode occurring after the age of 65 – has been consistently linked to increased subsequent incidence of dementia (Barnes et al., 2012; Diniz et al., 2013; Piras, Banaj, Porcari, Piras, & Spalletta, 2021; Santabárbara, Sevil-Pérez, Olaya, Gracia-García, & López-Antón, 2019). Recently, in a cohort study of over 300,000 participants, Yang et al. (2022) reported that a diagnosis of depression was associated with a 51% higher risk (HR, 1.51; 95% CI, 1.38–1.63; *p* < 0.001) of subsequently receiving a dementia diagnosis based on ICD-10 codes (Yang et al., 2022). This relationship is shown to be influenced by multiple factors including age, genotype, experience of early life adversity, age of onset, symptom duration, recurrence and severity of depression (Geerlings, Den Heijer, Koudstaal, Hofman, & Breteler, 2008; Ownby et al., 2006; Sachs-Ericsson et al., 2014; Singh-Manoux et al., 2017; Yu, Jung, Go, Park, & Ha, 2020). The Lancet report on *Dementia prevention, intervention, and care, 2020* notes that major depression accounts for 10% of the modifiable risk (Livingston et al., 2020). This, therefore, provides an opportunity to reduce dementia incidence by targeting MDD as a risk factor. Research suggests that improving mood symptoms in MDD can also alleviate cognitive deficits (Colwell et al., 2022). Yang et al. (2022) report that individuals older than 50 years who received pharmacological or psychological therapies for MDD, or a combination of both, were 30% less likely to develop dementia (HR 0.7, 95% CI, 0.62–0.77), as compared to those who did not undergo any such intervention (Yang et al., 2022). While this study excluded participants with treatment-resistant depression (TRD) from the analyses, another study showed that TRD enhances the risk of developing AD (Chan et al., 2020). Whilst the definition for TRD has not been standardised, for MDD, this may imply a minimum of two prior treatment failures and confirmation of prior adequate dose and duration (Gaynes et al., 2020). On this basis, up to one-third of individuals with MDD may be categorised as TRD and this poses significant challenges regarding inadequate or partial response to current antidepressant therapies (Sforzini et al., 2021). Therefore, treatment cessation, potentially due to lack of response or adverse drug reactions, may be associated with an increased risk of a subsequent dementia diagnosis as major depression is not adequately managed. The identification of shared pathological mechanisms underlying major depression and dementia, and development of new therapies that target these common pathways, may provide an approach to prevent dementia by treating depression.

### 2.2 The pathophysiological mechanisms underlying major depression and dementia

#### 2.2.1 Hippocampal atrophy is a common feature in major depression and early dementia

Structural magnetic resonance imaging (MRI) of neuroanatomical regions involved in mood regulation shows atrophy in those with MDD (Almeida, Burton, Ferrier, McKeith, & O'Brien, 2003; Gandy et al., 2017; Sampath et al., 2017; Sheline, Gado, & Price, 1998). Multiple areas within the limbic-cortical-striatal-pallidal-thalamic tract are affected (Sheline, 2000) and numerous studies find that hippocampal volume loss is consistently associated with clinical depression and correlates to hippocampal-dependent cognitive impairment (Gandy et al., 2017; Sampath et al., 2017). Further, hippocampal volume reduction is associated with number of depressive episodes and duration of symptoms, and may persist even after symptom resolution (Campbell, Marriott, Nahmias, & MacQueen, 2004; Frodl et al., 2002; Han et al., 2020; Nolan et al., 2020; Sapolsky, 2000; Schmaal et al., 2015; Sheline, Wang, Gado, Csernansky, & Vannier, 1996). This is particularly relevant as medial-temporal lobe atrophy of the hippocampus and entorhinal cortex are neuroimaging hallmarks of the earliest, observable stages of AD (Chauveau et al., 2021). Functional MRI (fMRI) indicates dysregulation in hippocampal-prefrontal cortex connectivity is a common factor in AD and major depression, and may potentially give rise to executive dysfunction and episodic-memory impairment seen in both conditions (Han et al., 2020; Liu et al., 2017; Sampath et al., 2017; Yang et al., 2021; Zhang, Peng, Sweeney, Jia, & Gong, 2018). Confounding factors such as disorder duration, severity and drug history make it difficult to distinguish between those with TRD and those responsive to treatment, but reduction in functional connectivity involving the default mode network (DMN) has been suggested (Barreiros et al., 2023; Runia et al., 2022); the relationship between DMN abnormalities specifically and cognitive impairment is unclear (Eyler et al., 2024). Recently however, differential patterns of functional connectivity were also observed between TRD and non-TRD participants and functional dysconnectvitiy in the same networks are associated with increased risk of progression to dementia (Lu et al., 2020; Sun et al., 2023).

#### 2.2.2 HPA axis dysfunction may drive inflammatory processes in neurodegeneration

Connections between the aforementioned regions and the hypothalamus mediate hypothalamic-pituitary-adrenal (HPA) (Herman et al., 2016). Central cortisol release is mediated by HPA axis activation as part of the stress response (Mikulska, Juszczyk, Gawrońska-Grzywacz, & Herbet, 2021). Pro-inflammatory cytokines are involved in stimulating glucocorticoid production via the HPA axis (Bellavance & Rivest, 2014; Jeon & Kim, 2016) and it is established that patients with major depression and dementia have increased levels of peripheral pro-inflammatory cytokines such as interleukin (IL)-1β, IL-6 and tumor necrosis factor (TNF)-α (Khemka et al., 2014; Ng et al., 2018). Both hyper- and hypocortisolaemia are seen in depressed patients, and prolonged HPA axis stimulation may lead to impaired negative feedback mechanisms influencing cortisol and cytokine production (Juruena, Bocharova, Agustini, & Young, 2018; Maripuu, Wikgren, Karling, Adolfsson, & Norrback, 2014; Nandam, Brazel, Zhou, & Jhaveri, 2020). Further, higher plasma concentration of inflammatory mediators such as TNF-α, IL-6 and high sensitivity CRP are associated with treatment failure in MDD contributing to TRD (Almutabagani et al., 2023; Haroon et al., 2018; Miller & Raison, 2016).

Further, the presence of pro-inflammatory cytokines, such as interferon-γ (IFN-γ), shift the metabolism of tryptophan to kynurenine and quinolinic acid, via indoleamine 2,3-dioxygenase (IDO) enzyme activity, instead of serotonin (5-HT) (Zunszain, Anacker, Cattaneo, Carvalho, & Pariante, 2011). Elevated levels of quinolinic acid are implicated in glutamatergic excitotoxicity, via overactivation of *N*-methyl-d-aspartate (NMDA) receptors, leading to oxidative stress and neuronal cell death (Marx et al., 2021); potentially impacting synaptic plasticity and influencing learning and memory. The increase in pro-inflammatory mediators leading to altered tryptophan metabolism concurrently results in 5-HT depletion, that may be associated with typical MDD symptoms, and may explain why some depressed patients benefit from conventional treatment with SSRIs and SNRIs as they block reuptake of 5-HT from the synaptic cleft via SERT inhibition thereby increasing its availability and activity. However, this may also explain why some patients do not respond to currently available medicines as reduced serotonin is a single aspect of complex mechanistic interplay; supporting critique of the reductive monoamine hypothesis of depression in recent years (Jauhar, Cowen, & Browning, 2023).

Glial cells, known as microglia and astrocytes, are non-neuronal cells that simultaneously release and are influenced by cytokines to support brain homeostasis (Vainchtein & Molofsky, 2020). Microglia are brain-resident macrophages with diverse functions in the developing and adult CNS (Li & Barres, 2017). In the pro-inflammatory hypothesis of depression, microglia respond to exogeneous Pattern-associated Molecular Patterns (PAMPs) or endogenous Damage-Associated Molecular Patterns (DAMPs) and secrete mediators, such as neurotoxic quinolinic acid (Steiner et al., 2011; Verdonk et al., 2019), and cytokines that lead to further recruitment of immune cells that may impact synaptic plasticity (Deng, Chen, & Wang, 2020; Wang et al., 2022a). Therefore, the activity of microglia is directly influenced by HPA axis dysfunction modulating inflammatory processes and may play a role in major depression and cognitive impairment.

#### 2.2.3 Dysregulated adult hippocampal neurogenesis and microglia are associated with depression and dementia

Adult hippocampal neurogenesis (AHN) is a dynamic mechanism that describes the creation and maturation of new neurons in the adult mammalian hippocampus. In response to various exogenous and subsequent endogenous mediators, hippocampal progenitor cells in the dentate gyrus proliferate and differentiate into neuroblasts or glial cells; immature neurons migrate as they mature throughout this process from the subgranular zone (SGZ) into the granule cell layer (GCL) to integrate with neuronal circuitry (Altman & Das, 1965; Kempermann et al., 2018; Kempermann, Song, & Gage, 2015; Spalding et al., 2013; Wu et al., 2023).

Since its discovery in rodents in the 1960s, AHN has been difficult to validate via neuroimaging techniques in the living human brain due to the absence of specific PET probes and the non-specific nature of MRI signal changes, which can be influenced by multiple biological processes (Altman & Das, 1965; Eriksson et al., 1998; Ho, Hooker, Sahay, Holt, & Roffman, 2013; Spalding et al., 2013). There are multiple extrinsic and intrinsic factors related to exercise, stress, ageing, sleep, nutrition, and pharmacological agents that may influence the rate of neurogenesis which do not necessarily correlate with dentate gyrus or hippocampal volume, or capture the temporal nature of stem cell proliferation and differentiation. Despite this controversy, growing evidence supports the presence of neural stem cells in the hippocampal niche that are able to produce neurons throughout life, which play an important homeostatic, rather than regenerative, role in neural plasticity (Boldrini et al., 2018; Lee & Thuret, 2018; Paredes et al., 2018; Sorrells et al., 2018; Spalding et al., 2013; Zhou et al., 2022). Whilst the precise role of AHN is yet to be determined, it is highly associated with spatial learning, pattern separation for memory encoding and retrieval, contextual fear conditioning, cognitive flexibility, and mood regulation; abnormal AHN is associated with both depressive symptoms and cognitive decline (Anacker et al., 2018; Anacker & Hen, 2017; Lucassen et al., 2019; Moreno-Jiménez, Terreros-Roncal, Flor-García, Rábano, & Llorens-Martín, 2021; Tartt et al., 2022; Tobin et al., 2019).

Microglia play a key role in controlling the number of new neurons produced in several complex ways (Diaz-Aparicio et al., 2020; Sierra et al., 2010). The hippocampus is a highly vascularised region which grants bloodborne factors direct access to the stem cell niche. In this way, microglia directly influence AHN by phagocytosing apoptotic newly born progenitor cells and synapses (Diaz-Aparicio et al., 2020; Sierra et al., 2010). Microglia not only respond to and release pro- and anti-inflammatory cytokines directly impacting neurogenesis, but also produce neurotrophic growth factors, such as brain-derived neurotrophic factor (BDNF) (Parkhurst et al., 2013). Such growth factors influence neuron growth, dendritic arborisation, and ultimately model synaptic architecture and plasticity in the hippocampus, and BDNF receptor polymorphisms are associated with depression and AD (Diaz-Aparicio et al., 2020; Turkin, Tuchina, & Klempin, 2021; Zhang et al., 2011). Serotonin also binds directly to microglia in the hippocampal niche to modulate neuroplasticity and therefore, pro-inflammatory mediators elevated in MDD leading to low levels of 5-HT and generation of neurotoxic species may be detrimental to AHN (Albertini et al., 2023; Alenina & Klempin, 2015; Helmut, Hanisch, Noda, & Verkhratsky, 2011; Marx et al., 2021; Pocock & Kettenmann, 2007; Sapolsky, 2000; Turkin et al., 2021; Zunszain et al., 2012). Further, in a multidirectional feedback loop, the hippocampus has a high density of glucocorticoid receptors (GR) and prolonged GR signalling mediated by excessive cortisol production, due to pro-inflammatory cytokine release by dysregulated microglia, may promote HPA axis dysfunction thereby impairing AHN (Anacker et al., 2013).

**Fig. 1:**
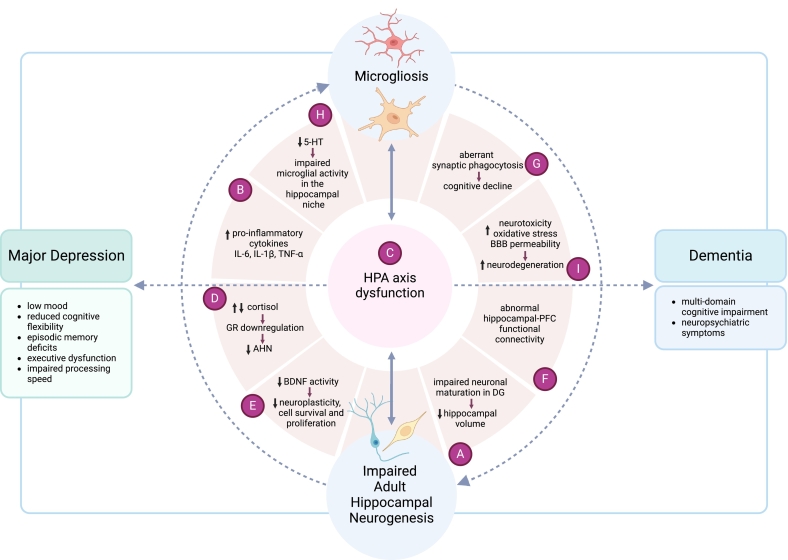
Potential overlapping mechanisms underlying major depression and dementia relating to impaired adult hippocampal neurogenesis and microglial activity. There is a complex relationship between major depression and dementia where cognitive dysfunction and neuropsychiatric symptoms may clinically manifest in both conditions suggesting an underlying common pathophysiology. Hippocampal volume loss, seen in MDD, may precede progressive neurodegeneration and cognitive decline in dementia (A). Increased levels of pro-inflammatory cytokines (B) may modulate HPA axis function (C) and microglial activity leading to glucocorticoid receptor downregulation (D), alterations in BDNF signalling and subsequent reduction of AHN (E). Microglia phagocytose apoptotic newborn neurons in the hippocampal niche. Newborn neurons in the dentate gyrus may also not become functionally mature, if BDNF impacted due to microgliosis, impacting hippocampal integrity and functional connectivity (A, F). Aberrant microglial activity may also lead to synapse uptake contributing to cognitive impairment (G). AHN may be influenced by low 5-HT levels (H) due to the presence of pro-inflammatory cytokines driving a shift in tryptophan metabolism leading to quinolinic acid production and further contributing to neurotoxicity in this region (I). (Created with BioRender.com)

Taken together, this indicates mechanisms connecting AHN and microglia, perhaps mediated by prolonged or inappropriate inflammatory processes, play a role in major depression and cognitive impairment (Fang et al., 2023; Herman, Simkovic, & Pasinetti, 2019) illustrated in Fig. 1. The downstream effect of innate immune system dysregulation may exacerbate inflammation and lead to subsequent neurodegeneration (Hayley, Hakim, & Albert, 2021; Herman, Simkovic, & Pasinetti, 2019). In a subpopulation of depressed patients, the lack of response to antidepressant therapies that do not sufficiently target these underlying mechanisms associated with disrupted AHN and microglial activity, may possibly contribute to TRD and increase risk of cognitive decline.

Crucially, longitudinal studies indicate pathophysiological changes in AD may occur decades before the onset of apparent cognitive decline (Beason-Held et al., 2013; Dubois et al., 2016; Jia et al., 2024; Parnetti, Chipi, Salvadori, D'Andrea, & Eusebi, 2019; Sperling et al., 2011). In these circumstances, major depression occurring during the prodrome may be regarded as a potential early indicator of neurodegenerative processes. (Maes et al., 2009; Dafsari and Jessen, 2020, Wiels, Baeken and Engelborghs, 2020). Therefore, focusing on MDD, especially late onset, to identify these molecular mechanisms may enable successful early diagnosis of preclinical dementia and subsequent intervention (Lee et al., 2021; Ownby et al., 2006; Siafarikas et al., 2021). Revealing biomarkers associated with pathological AHN and aberrant microglial activity in both MDD and neurodegenerative diseases may lead to the identification of potential drug targets for the development and discovery of modulatory compounds. This may also enable monitoring of treatment efficacy and disease progression.

## 3 Adult hippocampal neurogenesis in major depression and dementia

### 3.1 Adult hippocampal neurogenesis is directly impaired in major depression and dementia

While there may be an age-related decline in the rate of AHN (Tobin et al., 2019), *post-mortem* studies found differentially abnormal morphological development (related to morphometric qualities of the soma, cytoplasm and neurites) of DG granule cells in tissue derived from patients with probable AD, DLB, PD, FTD, amyotrophic lateral sclerosis (ALS) and Huntington's disease (HD) (Moreno-Jiménez et al., 2019; Terreros-Roncal et al., 2021). This suggests adult hippocampal neurogenesis is altered across several neurodegenerative diseases (Terreros-Roncal et al., 2021; Tobin et al., 2019).

In healthy controls (characterised based on clinical and neuropathological examination to exclude subjects with neurodegenerative disease history or markers, cognitive disability and brain cancer), radial glia-like (RGL) cells with neural stem cell properties, immature dentate granule cells and proliferative neuroblasts are present in the SGZ; indicating that neurogenic capacity of neural stem cells (NSCs) is preserved throughout life (Terreros-Roncal et al., 2021). Cells were identified at various stages of proliferation and differentiation, using markers such as SRY-Box Transcription Factor 2 (SOX2) and neuroepithelial stem cell protein (Nestin), which are indicative of stem cell-like properties, in addition to polysialylated-neural cell adhesion molecule (PSA-NCAM), neuron-specific doublecortin (DCX) and Prospero homeobox protein 1 (PROX1), which are expressed by immature dentate granule cells (DGCs) (Terreros-Roncal et al., 2021).

In tissue derived from ALS, HD and PD patients, there was an increased density of RGLs and DCX+ immature DGCs, of which a higher proportion were associated with aberrant morphology; where DGCs remained undifferentiated for longer in HD. Despite both being caused by α-synuclein, PD and DLB produced different AHN signatures which may be associated with the trajectory of cognitive impairment which usually differs between the conditions (Terreros-Roncal et al., 2021). In PD, there was an increase in HuC/HuD+ proliferating neuroblasts and reduced neuronal nuclei (NeuN) expression on DCX+ immature DGCs suggesting impaired neuronal maturation compared to DLB which also showed, albeit milder, alterations in AHN. Conversely in FTD, there is a reduction in HuC/HuD+ proliferative neuroblasts, moderately impaired DGC differentiation and an imbalance in the ratio of RGLs to proliferating cells (Terreros-Roncal et al., 2021). The age and extent of hippocampal atrophy at time of death may contribute to these observed differences. In line with other neurodegenerative diseases, Jin et al. (2004) found that DCX+ and PSA-NCAM were highly expressed in AD compared to controls indicating increased neuroproliferation (Jin et al., 2004). However, Moreno-Jiménez et al. (2019) noted a steep decline in density and neuronal maturation relative to AD progression (Moreno-Jiménez et al., 2019).

Similarly, microstructural alterations in the dentate gyrus may be predictive of MDD development (van Dijk et al., 2021). Analyses of *post-mortem* tissue from depressed patients align with imaging studies revealing reduction in GCL volume, as well as reduced numbers of NeuN+ and Nestin+ cells in the anterior DG (Berger, Lee, Young, Aarsland, & Thuret, 2020). In the ventral dentate gyrus (vDG) in mice, which corresponds anatomically to the anterior DG in humans, functional neurogenesis is strongly associated with stress resilience and cognitive flexibility, often impaired in depression (Anacker et al., 2018; Anacker & Hen, 2017). This suggests DG morphological changes impacting adult hippocampal neurogenesis play an important role in the pathophysiology of major depression. Furthermore, this putative reduction in the rate of adult hippocampal neurogenesis may manifest as episodic memory deficits in MDD (Fang, Demic, & Cheng, 2018); supporting the use of depression as a prodromal sign and potential stratification tool in neurodegenerative diseases.

Multiple *post-mortem* studies indicate that the rate of AHN is altered in AD patients in the early stages of the disease (Li et al., 2008; Zhou et al., 2023); however, there is a discrepancy in the directionality (Boekhoorn, Joels, & Lucassen, 2006; Tobin et al., 2019). Terreros-Roncal et al. (2021) suggest that the nature of AHN may enhance vulnerability to neurodegeneration underlying hippocampal dysfunction which occurs during physiological and pathological ageing (Terreros-Roncal et al., 2021); this may be exacerbated in the context of MDD. In neurodegenerative diseases, although there is an increased number of NSCs produced, they do not become functionally mature which may impact the integrity of hippocampal circuitry influencing cognitive impairment.

Despite the challenges measuring AHN *in vivo* in humans, *in vitro* assays modelling hippocampal neurogenesis correlates are useful for capturing the temporal variability associated with AHN and may provide insight into changes in neurogenesis that occur in those living with MDD and also during the preclinical phase of AD. Du Preez et al. (2022) used a parabiosis assay of foetal-derived hippocampal progenitor cells to determine variation in AHN biomarkers in those with and without depressive symptomatology (Du Preez et al., 2022). They found that serum from people with recurrent depressive symptoms induced impaired neuronal morphology and depression symptoms in later life was associated with changes in apoptosis and differentiation (Du Preez et al., 2022). Using the same assay, Borsini et al. (2019) investigated the effect of IFN-α treatment in Hepatitis C patients who developed major depression (Borsini et al., 2019), they observed greater apoptosis during the HPC proliferation phase and reduction in DCX+ expressing immature neurons during differentiation. Based on changes in these markers, they could predict onset of depression after 4 weeks of IFN-α treatment, highlighting the potential impact of innate immune system and pro-inflammatory markers on AHN. Again, using this model as a proxy biomarker in a longitudinal study, Maruszak et al. (2023) looked at individuals with MCI who developed AD and those who did not (Maruszak et al., 2023). Markers of neurogenesis during proliferation and differentiation, defined by stem cellness (SOX2, Nestin), proliferation (Ki67), neuronal maturation (DCX, MAP2) and cell death (CC3), were assessed at multiple timepoints after HPC exposure to MCI patients' serum. These markers were related to cognitive function score by MMSE. They observed increased proliferation of DCX+ cells during differentiation suggesting increased neurogenesis in MCI-converters prior to diagnosis. In this study, neurogenic readouts Ki67 + % and average cell number during proliferation and CC3 + % during differentiation (in addition to years in education) enabled prediction of progression to AD from MCI.

Further, using the HPC cell line in the above assays, Borsini, Di Benedetto, Giacobbe, and Pariante (2020) exposed the cells to high concentrations of IL-6 and IL-1β which led to a decrease in neurogenesis suggesting pro-inflammatory mediators, if released by microglia, may have a direct effect on AHN (Borsini et al., 2020). Collective evidence suggests that alterations in AHN may serve as a potential biomarker for early disease detection before there is significant cognitive impairment. Although a clear AHN-AD signature has not yet been characterised, animal models investigating the impact of AD risk-factor or causative genes, including APP, PSEN1 and Apolipoprotein E (APOE) ε4, indicate that AD pathology is implicated in dysfunctional AHN (Crews et al., 2010; Donovan et al., 2006; Feng et al., 2001; Haughey et al., 2002; Wang, Dineley, Sweatt, & Zheng, 2004; Zhang, McNeil, Dressler, & Siman, 2007).

### 3.2 Mechanisms by which dysregulated microglia may impair adult hippocampal neurogenesis in major depression and dementia

Pathological inflammatory processes mediated by microglia are a hallmark of AD and associated with several neurodegenerative diseases (Guan et al., 2022; Xu et al., 2016; Xu, Jin, Yang, & Jin, 2021; Zhang, Wang, Hu, Zhao, & Sun, 2021). While the role of microglia on AHN in MDD is yet to be fully established (Deng et al., 2020; Fan et al., 2022; Fang et al., 2018; Wang et al., 2022a), in *post-mortem* studies that show AHN is impacted in neurodegenerative diseases, the common thread in all tissues was increased pyknosis, impaired microglial phagocytic capacity, varying degrees of astrogliosis and altered microvasculature in the dentate gyrus (Essa, Peyton, Hasan, León, & Choi, 2022; Terreros-Roncal et al., 2021). In unmedicated individuals with MDD, PET studies suggest an increase in glial density indicated by Translocator protein 18 kDa (TSPO), a putative biomarker of inflammation (Richards et al., 2018). However, in MDD *post-mortem* research profiling microglia using single cell analysis, findings do not suggest pro-inflammatory morphology but rather microglia may have enhanced homeostatic functions (Snijders et al., 2020). An increase in P2Y_12_ receptor, TMEM119 and CCR5 (CD195) expression observed in the frontal lobe, temporal lobe, thalamus, and subventricular zone of medicated individuals with MDD corroborates this (Böttcher et al., 2020). While the hippocampus was not examined in these studies, issues regarding tissue processing may affect microglial phenotype and yield conflicting results; including auto-fluorescence and ante- and peri-mortem factors, such as antidepressant use (Nicolai, Nettesheim, de Witte, & Snijders, 2023). This contrasts both clinical and pre-clinical evidence suggesting that microgliosis in multiple brain regions, including the hippocampus, is associated with depressive-like behaviours (Butovsky et al., 2006; Duman & Aghajanian, 2012; Fan et al., 2022; Fang et al., 2023; Wang et al., 2022b; Zhang, Zhang, & You, 2018). Therefore, the potential role of microglia on AHN in MDD requires further investigation. Despite this, serum pro-inflammatory markers such as IL-6, TNF-α and IFN-γ are significantly higher in those with MDD who have a CRP level 3 mg/L (Sforzini et al., 2023). Regardless of level of peripheral CRP or other proteins, mRNA expression analyses show an upregulation of pro-inflammatory and glucocorticoid-related genes such as A2M, CCL2, IL-1β, IL-6, MIF, FKBP5, SGK1, STAT1 and TNF-α, whilst glucocorticoid receptor is downregulated suggesting innate immune system dysregulation. GR downregulation is associated with reduced AHN and pro-inflammatory cytokines, released by and acting on microglia, may influence BBB permeability and integrity and potentially drive pathogenesis in AD (Wang, Tan, Yu, & Tan, 2015).

In AD, Aβ-42 production and clearance is disrupted leading to aggregated Aβ plaques, though it is not clear whether this mechanism is causative or consequential pathologically as amyloid and tau burden does not necessarily correlate with cognitive function status (Hsu et al., 2015; Villemagne et al., 2013; Ziontz et al., 2019). Recently, AD has been postured as an autoimmune condition whereby soluble Aβ may act as, and is produced in response to the detection of, DAMPs triggering an innate immunity cascade (Clark & Vissel, 2015; Meier-Stephenson et al., 2022; Weaver, 2022). Generally, aggregated, intracellular Aβ-42 plaques and extracellular dephosphorylated neurofibrillary tau tangles (NFTs) may exacerbate inflammatory processes, leading to stimulation of surveillant microglia and polarising them to a pro-inflammatory morphological state (De et al., 2019; Perea, Bolós, & Avila, 2020).

Notably, microglial-associated mutations in triggering receptor expressed on myeloid cells 2 (TREM2), APOE, CD33 and CR3 significantly heighten AD risk (Wolfe, Fitz, Nam, Lefterov, & Koldamova, 2019). *Post-mortem* studies reveal that primary human adult microglia phagocytose AD patient-derived synapses to a greater extent, compared to controls (Rajendran & Paolicelli, 2018). This may be a protective mechanism initially, as synapses may become apoptotic when exposed to Aβ oligomer and are engulfed by microglia due to TREM2 signalling to resolve synaptic hyperactivity in an *in vivo* hAPP NL-F knock-in mouse model of AD (Rueda-Carrasco et al., 2023). This implies that altered synaptic uptake by microglia, due to TREM2 loss of function for example, is an important mechanism associated with cognitive decline as it corresponds to synapse loss (Hong et al., 2016; Rajendran & Paolicelli, 2018; Tzioras et al., 2019).

Microglia also produce neurotrophic factors such as BDNF. Alterations in serum BDNF and BDNF gene polymorphisms are associated with both MDD and cognitive decline (Borroni et al., 2009; Hall et al., 2011; Martinowich, Manji, & Lu, 2007; Porter & O'Connor, 2022; Saral et al., 2023; Wetsel et al., 2013; Yang et al., 2020; Zhang et al., 2011). Single nucleotide polymorphisms of BDNF 196 A allele have been identified in AD patients with major depression and may influence response to antidepressant paroxetine (Zhang et al., 2011). Philpotts, Gillan, Barrow, and Seidler (2023) find reduced hippocampal BDNF expression is associated with MDD in post-mortem studies and in preclinical models of a depressive-like phenotype induced by chronic stress (Philpotts et al., 2023). Further, Liu, Li, Su, Wang, and Jiang (2019) posit that dysregulation of microglial pro- and anti-inflammatory states in the hippocampus confer depressive-like behaviours in mice which may be mediated by decrease in BDNF-TrkB activity (Liu et al., 2019). Specifically, lower hippocampal BDNF levels are associated with reduced neuroplasticity and correlate with MDD and cognitive impairment (Yang et al., 2020).

Therefore, gene risk variants that compromise microglial activity related to cytokine production, survival, proliferation, and phagocytosis may exacerbate Aβ plaque and NFT deposition by failing to reduce clearance of pathological proteins (Perea et al., 2020). In a vicious circle, the resultant disruption in cellular processes and synaptic function leads to cell death, oxidative stress and production of reactive oxygen species stimulating further pro-inflammatory processes, mediated by aberrant microglial activity, compounding neuronal loss. The overall consequence of this chronic inflammatory response leads to BBB degradation, dysregulated neurotransmitter function and neurodegeneration affecting multiple domains which may clinically present as mood and cognitive deficits in depression and may be driving cognitive dysfunction in dementia (Santos et al., 2016).

Taken together, the current evidence suggests that dysregulated AHN, potentially influenced by irregular microglial activity, may be a convergent mechanism underlying dementia and MDD (Berger et al., 2020). Compounds capable of restoring homeostasis in these pathological processes may not only be beneficial for symptomatic cognitive impairment and depression but may also have disease modifying potential in neurodegenerative diseases.

### 3.3 The impact of antidepressant and immunomodulatory agents on mood and cognitive function via AHN and microglia

Antidepressants may increase AHN and elucidating their mechanism of action may aid identification of pathomechanisms and potential biomarkers correlating to MDD and cognitive impairment (Chen et al., 2007; Mahar, Bambico, Mechawar, & Nobrega, 2014; Planchez et al., 2021). Antidepressants that inhibit monoamine uptake or reuptake may restore hippocampal neuroplasticity and possibly have neurodegenerative disease modifying potential (Andrade & Kumar Rao, 2010). It has been previously hypothesised that citalopram (an SSRI) may impact amyloid precursor protein (APP) processing and reduce Aβ plaque load in a human-derived neural stem cell model with fAD PSEN1 mutation (Elsworthy et al., 2022). However, the effect of SSRIs on cognitive function in both depressed patients and those with AD is difficult to determine. Vortioxetine (an SSRI) may improve executive dysfunction in MDD but the data are inconsistent (McIntyre, Harrison, Loft, Jacobson, & Olsen, 2016; Pehrson, Li, Sanchez, & Gulinello, 2021). In the context of staving off cognitive decline, SSRIs do not appear to show clear significant benefit (Bouter & Bouter, 2022; Chow, Pollock, & Milgram, 2007; Mdawar, Ghossoub, & Khoury, 2020; Schulkens et al., 2022). A multitude of complex factors however, including degree of brain atrophy at baseline, treatment dose and duration, methodological design and heterogeneity across studies, may explain these results. Some licensed antidepressants, such as TCAs, MAOIs and some SSRIs have anticholinergic effects and are associated with cognitive impairment and paradoxically, both increased and decreased dementia risk (Moraros, Nwankwo, Patten, & Mousseau, 2017; Pieper et al., 2020; Saczynski et al., 2015; Wang et al., 2018). Therefore, antidepressant class and time of intervention may influence the trajectory of neurodegeneration.

Structural MRI indicates that in people with depressive episodes who took antidepressants for over three years, a significant increase in hippocampal volume was observed and this predicted a better clinical outcome (Frodl et al., 2002). Histological analysis of *post-mortem* brain tissue from MDD patients treated with SSRIs and TCAs suggests that the proportion of NPCs and angiogenesis were increased in the anterior and mid-DG, which corresponded to higher DG volume, compared to those untreated, relative to controls (Boldrini et al., 2009; Boldrini et al., 2012). Overall, MDD patients who had received antidepressant treatment had more GNs but the same number of NPCs which may suggest the mechanism of action modulates impaired cell survival and maturation, rather than proliferation (Boldrini et al., 2013); perhaps mediated by increasing vascularisation. While a younger age of MDD onset correlates with decline in aDG GN number, which may increase with antidepressant therapies, this may not occur in older adults. Lucassen et al. (2010) found no significant differences in NPCs or proliferating cells in SGZ and GCL derived from MDD patients treated with SSRIs, TCAs or MAOIs, compared to those untreated, relative to non-depressed controls (Lucassen et al., 2010). This may be related to variability in causes of death, duration of concomitant medication, such as antihypertensives, which may influence AHN, plus age of onset, duration and severity of MDD (De Lucia et al., 2022). From these studies, no immunohistochemical differences were observed between groups in the posterior DG making it difficult to speculate the impact of antidepressants on cognitive function. However, tissues from subjects are carefully selected to exclude neurological conditions so changes observed may not be relevant to a specific subset of people who experience depression with cognitive deficits. Although in PDD/DLB patients, Gatt et al. (2017) found SSRI use was associated with higher DCX+ expressing cells in the SGL which was related to preserved cognitive function (Gatt et al., 2017).

Santarelli et al. (2003) postulate that AHN-related behavioural effects of fluoxetine (an SSRI) may be mediated by 5-HT1a receptors (Santarelli et al., 2003). In mice, fluoxetine increased BrdU+ labelled DG progenitor cells corresponding to increased neurogenesis after 11+ days treatment improving depression-like behaviour, which was not seen in 5HT1a knockout mice. Similarly, David et al. (2009) used corticosterone to induce a depression and anxiety-like phenotype in rodents and found that fluoxetine increased neuronal proliferation, differentiation, and survival correlating with behavioural effects, but may not target hippocampal neurogenesis mechanisms solely. In this chronic stress model, fluoxetine did not impact stress-induced blunted corticosterone elevation, suggesting these drugs do not directly modulate HPA-axis activity to influence AHN (David et al., 2009). In a human cellular model, reduction in hippocampal progenitor cell proliferation and differentiation induced by dexamethasone was rescued by sertraline. This suggests antidepressants may mediate AHN via glucocorticoid receptor activation in the neurogenic niche (Anacker et al., 2011).

It is possible that potential beneficial effects of certain antidepressants on AHN do not address the multifaceted, pathophysiological commonalities between MDD, particularly in a treatment-resistant population, and neurodegenerative diseases. It is therefore difficult to draw meaningful conclusions on the benefit of these drugs on cognitive function or dementia risk. Thus, new or existing compounds that address the multitude of intersecting pathological mechanisms underlying a pro-inflammatory subtype of MDD potentially accelerating neurodegeneration, delivered at an appropriate time in the disease course, may preserve cognitive function.

Minocycline, a broad-spectrum tetracycline antibiotic that modulates the tryptophan-kynurenine pathway (TKP), IL-1β, IL-6 and TNF-α levels, and microglial phagocytosis demonstrates promise for targeting neuroinflammatory mechanisms in neurodegeneration. This has been associated with ameliorating depressive-like behaviours and cognitive function (Bassett et al., 2021; Cheng et al., 2023; Panizzutti et al., 2023; Poggini et al., 2023). In a placebo-controlled trial in patients with mild AD however, 200 mg and 400 mg minocycline did not significantly delay cognitive or functional decline after two years of treatment (Howard et al., 2020). Several factors may have contributed to this outcome relating to high drop-out rate and low compliance. Poor tolerability in the 400 mg treatment-arm due to gastrointestinal and dermatological side effects suggests it may not be clinically feasible or appropriate to investigate the efficacy of higher doses in this vulnerable population. It is not clear whether neuroinflammatory markers were assessed at baseline to ascertain if modulated by minocycline. The mean participant age was 74 years, with established mild cognitive impairment confirmed by sMMSE, implying the intervention may have been applied at too late a stage and at a potentially sub-therapeutic dose to effectively modulate the disease trajectory.

Rather, lending credence to the autoimmune hypothesis of AD, a case-control study found that use of methotrexate, an immunosuppressive agent, was associated with a lower risk of developing dementia in patients with rheumatoid arthritis and may potentially mitigate suicidal ideation in this patient population (Newby et al., 2020; De Oliveira et al., 2013). This suggests that administration of immunomodulating drugs, with multi-directional effects on Aβ-42 and serotonin, during the preclinical phase of disease may be capable of modifying neurodegenerative trajectory to influence mood and cognitive function; potentially mediated by microglial dysfunction and impacting AHN.

## 4 Psilocybin

### 4.1 The effect of psilocybin on mood and cognitive function

Psilocybin is a compound naturally derived from *psilocybe* containing mushroom species which have held medicinal, cultural, and spiritual importance, with use well-documented in Mesoamerica, for thousands of years (Carod-Artal, 2015). In the last decade, there has been a resurgence in the interest of the therapeutic potential of psychedelics after research was restricted in the 1970s (Doblin, Christiansen, Jerome, & Burge, 2019).

Oral administration of a therapeutic dose of psilocybin, usually ranging from 0.3 to 0.6 mg/kg or fixed at 25 mg (Dahmane, Hutson, & Gobburu, 2021), produces acute perceptual alterations creating an experience that may lead to meaningful insights and contribute to improving mood (Davis et al., 2021; Davis, Barrett, & Griffiths, 2020; Griffiths, Richards, Johnson, McCann, & Jesse, 2008). Several Phase II randomized controlled trials (RCTs) in patients with MDD and TRD demonstrate that single- or double-dose 25 mg psilocybin, combined with psychotherapy, produces a fast and sustained antidepressant response, when compared to lower doses of psilocybin, and may be as effective as SSRI, escitalopram (Carhart-Harris et al., 2021; Goodwin et al., 2023; Raison et al., 2023).

Several studies suggest psilocybin enhances cognitive flexibility, which augments psychotherapy for MDD and it has been proposed this is mediated by adult hippocampal neurogenesis (Daws et al., 2022; Doss et al., 2021; Magaraggia, Kuiperes, & Schreiber, 2021). Doss et al. (2021) reported increasing cognitive and neural flexibility following two doses of 20 mg and 30 mg psilocybin (per 70 kg person) in MDD patients (Doss et al., 2021). They assessed executive function set-shifting against magnetic resonance spectroscopy (MRS) glutamatergic and *N*-acetylaspartate (NAA) neurotransmission but did not observe a relationship between changes in depressive symptoms and cognitive flexibility. Unfortunately, they were not able to assess hippocampal functional connectivity due to signal variability. Rucker et al. (2022) assessed the effects of 25 mg and 10 mg psilocybin, compared to placebo, on cognitive function via the Cambridge Neuropsychological Test Automated Battery (CANTAB) in healthy adults (Rucker et al., 2022). In those treated with psilocybin 25 mg, they observed a reduction in episodic memory measure, Paired Associates Learning (PAL) score which may indicate better performance, but this was not statistically significant. There was, however, an increasing trend in global composite scores in both 25 mg and 10 mg psilocybin groups as compared to baseline, but not to placebo. Limitations relating to small sample sizes, tasks utilised and assessment in healthy participants, make it difficult to draw conclusions about the effect of psilocybin on hippocampal-dependent cognitive function in humans. It is possible that differences may only be notable in patient populations where there is baseline pathophysiological dysfunction.

Naturalistic studies and data regarding recreational or illicit use of psilocybin have not been considered in this review due to confounding variables associated with dose, frequency of administration, and concomitant use of psychoactive substances that would prevent inferring the sole effect of psilocybin on mood, cognitive function and dementia risk.

### 4.2 The impact of psilocybin on adult hippocampal neurogenesis

#### 4.2.1 Psilocybin directly modulates adult hippocampal neurogenesis via neuroplasticity in preclinical models

The antidepressant properties of psilocybin are associated with promoting neuroplasticity which may have direct relevance to modulating adult hippocampal neurogenesis (Olson, 2018). In an animal model relevant to post-traumatic stress disorder (PTSD), Du et al. (2023) report that single-dose 2.5 mg/kg psilocybin facilitates rapid and persistent fear extinction in fear-conditioned mice (Du et al., 2023). They observe a greater proportion of DCX+ and BrdU+ cells in the DG 7 days after psilocybin administration, compared to vehicle-treated fear-conditioned mice. They note that psilocybin significantly reduced freezing time and rescued a decrease in hippocampal dendritic plasticity, characterised by branches and spine density. This coincides with the reversal of decreases in BDNF and mTOR expression in fear-conditioned mice treated with psilocybin. Similarly, Catlow, Song, Paredes, Kirstein, and Sanchez-Ramos (2013) also report 0.1 mg/kg psilocybin numerically increased the number of BrdU+/NeuN+ hippocampal progenitor cells, although this was not statistically significant relative to vehicle controls. Notably, higher doses of 0.5 mg/kg and 1 mg/kg psilocybin decreased BrdU+ neural progenitor cell survival in a dose-dependent manner, the effects of which were blocked by non-selective 5-HT receptor (5-HTR) antagonist, ketanserin (Catlow et al., 2013). These data emphasise the importance of dose-finding experiments to determine the therapeutic window of psilocybin for optimally modulating AHN.

Psilocybin undergoes first pass metabolism via the liver where it is rapidly dephosphorylated to main active metabolite, psilocin a serotonin analogue. Psilocin is able to cross the blood brain barrier (BBB) where it acts as an agonist at 5-HT2a receptors in the cortex; these receptors are expressed in the hippocampus and notably DG (Burnet, Eastwood, Lacey, & Harrison, 1995; Peddie, Davies, Colyer, Stewart, & Rodríguez, 2008). There is, however, evidence that it may bind other 5-HT receptor subtypes, receptor classes and monoamine transporters to a lesser extent (Blough et al., 2014; Dinis-Oliveira, 2017; Erkizia-Santamaría, Alles-Pascual, Horrillo, Meana, & Ortega, 2022a; Halberstadt & Geyer, 2011). For example, Vargas et al., 2023a, Vargas et al., 2023b posit that lipophilic properties of psilocin enable passage through the cell membrane to activate intracellular 5-HT2a G-protein coupled receptors (GPCRs); distinguishing its activity from that of serotonin in the context of promoting neuroplasticity (Vargas et al., 2023b).

Further, psilocin is also an agonist at 5-HT1a receptors, present on mature GCs in the DG, which may be key for modulating its antidepressant activity influenced by BDNF (Hensler, Advani, & Monteggia, 2007; Pokorny, Preller, Kraehenmann, & Vollenweider, 2016; Segi-Nishida, 2017). TCB-2 (4-Bromo-3,6-dimethoxybenzocyclobuten-1-yl)methylamine hydrobromide), a selective 5HT2aR agonist, and NAD-299, a 5-HT1aR antagonist, decreased oxidative stress and reduced hippocampal neuronal loss induced by streptozotocin in an AD model in male Wistar rats (Afshar, Shahidi, Rohani, Soleimani Asl, & Komaki, 2019). This may suggest a potential role for psilocin, via shared mechanisms of action, in mitigating oxidative damage preceding neurodegeneration via modulation of these receptors. However, Hesselgrave, Troppoli, Wulff, Cole, and Thompson (2021) observe psilocybin 1 mg/kg strengthens hippocampal excitatory synapses correlating with reduced anhedonic behaviour in stressed mice, the effects of which were not attenuated by ketanserin, suggesting 5HT-independent mechanisms also at play (Hesselgrave et al., 2021).

Ly et al. (2018) demonstrated that 5HT2aR activation leads to stimulation of tropomyosin receptor kinase B (TrkB), the primary receptor for BDNF, and mammalian target of rapamycin (mTOR) signalling pathways resulting in prominent changes in rat cortical neurons such as increased neurite outgrowth, dendritogenesis, spinogenesis and synaptogenesis (Ly et al., 2018). Independent of 5-HT, Moliner et al. (2023) found that psilocin may act as a positive allosteric modulator of BDNF by binding to the extracellular transmembrane domain of TrkB leading to a conformational change to enable favourable binding by BDNF, the concentration of which is already increased; thereby enhancing BDNF signalling and downstream effects of TrkB activation (Moliner et al., 2023). Although not assessed with psilocybin, LSD increases long-term neuronal survival of dentate granule cells (DGCs) in the hippocampus 4 weeks after single dose administration in mice; the effects of which are mediated by TrkB binding as established with psilocin (Moliner et al., 2023). Psilocin also binds to TrkB with greater affinity than fluoxetine which may explain its rapid activity compared to other antidepressants. This suggests the neuroplastic effects of psilocybin which mediate antidepressant activity may also play a role in protecting against neurodegeneration by potentially stimulating neurogenesis in the hippocampus and influencing networks impacted in cognitive impairment.

Psilocin also exhibits biased agonism whereby ligand binding to 5-HT2aRs results in selective activation of signal transduction pathways mediated by G-proteins or β-arrestins (López-Giménez & González-Maeso, 2018). The associated Gq-phospholipase signalling pathway typically leads to endoplasmic reticulum Ca2+ release induced by inositol 1,4,5-trisphosphate (IP3) and activation of protein kinase C (PKC). At the Gi-GPCR 5-HT1aR, psilocin may also be a partial agonist and inhibit adenylyl cyclase activity leading to reduction in Ca2+ mediated by PKA (Halberstadt & Geyer, 2011; Smausz, Neill, & Gigg, 2022). The impact of this differential cellular response results in varied physiological and hippocampal processes affecting spatial learning and working memory, and it is not yet clear what factors influence which cascade in order to predict acute or chronic response (Carter et al., 2005; Zhang & Stackman, 2015).

#### 4.2.2 Psilocybin may indirectly influence adult hippocampal neurogenesis via immune modulation

Psilocin has strong affinity for 5-HT2bR (Ki = 4.6 nM), the main 5-HT receptor subtype expressed by microglia, and may bind, with variable affinity, to 5-HT receptor subtypes 1 A, 1D, 2 A, and 2C (Albertini et al., 2023; Krabbe et al., 2012; Passie, Seifert, Schneider, & Emrich, 2002). Therefore, it may be theoretically plausible for psilocin to modulate activity via serotonin receptors on microglia to influence AHN. However, dosing regimens to ensure safety must be developed as 5HT2bR activation may be associated with cardiac valvulopathy when “microdosed” (at a sub-threshold dose for subjective acute effects) (Hutcheson, Setola, Roth, & Merryman, 2011). Whilst the evidence for an effect of psilocin on microglia form and function is thus far, limited, known 5-HT2a agonists demonstrate ability to modulate immune cell functions and may be anti-inflammatory (Galvão-Coelho et al., 2020; Szabo, Kovacs, Frecska, & Rajnavolgyi, 2014). Preliminary studies in the grey literature show potential immunomodulatory effects of psilocybin (Inserra, De Gregorio, & Gobbi, 2021; Kozłowska, Klimczak, Wiatr, & Figiel, 2021; Mason et al., 2022; Szabo, 2015; Thompson & Szabo, 2020; VanderZwaag et al., 2023).

The effects of psilocybin on microglia may lead to the release of, or be modulated by, immune mediators in the blood. In a placebo-controlled study in healthy participants, blood samples were collected at baseline, between 1 and 6 h and at 7 days post-administration of 0.17 mg/kg psilocybin (12 mg for 70 kg person) or placebo. Immediate reduction in TNF-α and increase in cortisol were observed (Mason et al., 2023). Holze et al. (2022) also observe, acutely, a significant increase in plasma cortisol, and non-significant increase in plasma BDNF, following 15 mg or 30 mg psilocybin administration in healthy participants (Holze et al., 2022). In the study conducted by Mason et al. (2023), at Day 7, TNF-α returned to baseline whilst IL-6 and CRP levels had significantly reduced compared to placebo. No significant changes were seen in the concentrations of IL-8 or IL-1β. Seven days after drug administration, participants who underwent the Maastricht acute stress test (MAST) to induce an autonomic, glucocorticoid-dependent and subjective stress response experienced an increase in cortisol, compared to those who did not complete the stress test, regardless of treatment group. Remarkably, psilocybin appeared to diminish the stress response by dampening cortisol increase. Further, lower TNF-α levels correlated with reduced hippocampal glutamate modulation, measured via MRS, and sustained positive improvement in mood corresponding to reducing IL-6 and CRP concentrations (Mason et al., 2023). This data suggests psilocybin may modulate HPA-axis stress response via attenuation of glutamatergic transmission in the hippocampus.

Conversely, a study of 16 healthy participants measuring CRP, TNF-α and soluble urokinase plasminogen activator receptor (suPAR) did not find psilocybin, at a mean dose of 0.22 mg/kg, to be anti-inflammatory (Burmester et al., 2023). Consistent with the study conducted by Mason et al. (2022), TNF-α increased but not significantly. However, blood samples were taken at baseline and one day after drug administration which may likely not be enough time to see persistent changes if any, in these markers once the acute phase passes. Additionally, markers were not assessed relative to behavioural outcomes and participants were part of different neuroimaging studies under variable conditions where some received doses between 0.2 and 0.3 mg/Kg, doses ranging between 3 and 30 mg or a fixed 18 mg dose and data may not be directly comparable (Madsen et al., 2019, Madsen et al., 2020; McCulloch et al., 2022; Søndergaard et al., 2022). The major limitations of both studies relate to small sample size, limited number of inflammatory markers assessed, and use of sub-acute doses in healthy participants; immune modulation may be dose- and baseline-dependent where changes may only be notable in people with higher levels of pro-inflammatory cytokines or dysregulated HPA-axis response. Robust studies, accounting for confounding variables, are needed to assess the impact of psilocybin on neuroinflammatory marker changes in patient populations at varying doses relative to acute and longitudinal outcomes for mood and cognitive function. In 57BL/6 male mice, 3 mg/Kg intraperitoneal psilocybin induced anxiolytic-like effects 4 h post-administration and persisted for 7 days. This effect was not diminished by pre-treatment with a 5-HT2a antagonist but was attenuated with mifepristone, a glucocorticoid receptor antagonist, as well as when psilocybin-induced corticosterone elevation was suppressed (Jones et al., 2023) The collective in vitro and in vivo pre-treatment data suggests that psilocybin may provide resilience to stressors indicating neuroprotective potential, but further research is needed to ascertain this.

These effects may be mediated by psilocybin-induced cytokine modulation. In vitro studies suggest that human macrophage cell line, U937, treated with mushroom-derived psilocybin-containing water extracts, after exposure to LPS, resulted in inhibition of TNF-α and IL-1β production and a significant reduction in IL-6 and cyclooxygenase-2 (COX2) (Nkadimeng, Steinmann, & Eloff, 2021). Further, when mice pre-treated with 0.88 mg/kg psilocybin were injected with LPS, mRNA expression of COX2 and TNF-α was significantly downregulated. In mice administered psilocybin after LPS injection, only TNF-α was reduced (Zanikov et al., 2023). However, it may be difficult to assess impact of psilocybin on HPA axis activation or pro-inflammatory markers in animal models where stress due to researcher handling or environment may be difficult to regulate; unintendedly influencing behaviour, biological response, and potentially misrepresenting drug effects.

**Fig. 2:**
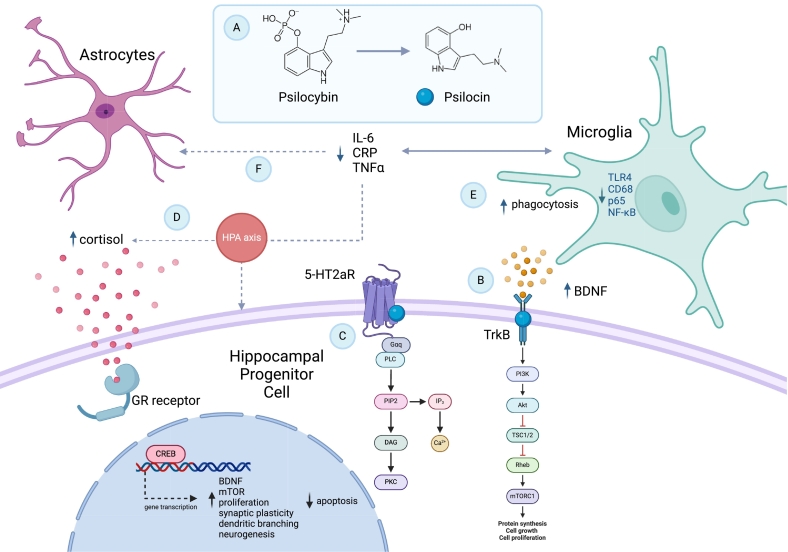
Proposed mechanism of action of psilocybin involving different cell types in the hippocampal niche. (A) Psilocybin is dephosphorylated to active metabolite, psilocin. (B) In vitro, psilocin increases BDNF concentration while simultaneously enhancing its activity via positive allosteric modulation of tropomyosin receptor kinase B (TrkB) (Moliner et al., 2023) and may potentially influence mTOR signalling (Ly et al., 2018), to promote neuroplasticity. Further, TrkB binding is associated with increasing hippocampal DGC survival and neurogenesis relative to attenuating depressive-like behaviours in mice (Moliner et al., 2023). Supporting this, Du et al. (2023) also report increased proportion of DCX+ and BrdU+ cells in the dentate gyrus 7 days post-psilocybin administration, corresponding to higher concentrations of BDNF and associated with fear-extinction in mice (Du et al., 2023). (C) Hippocampal progenitor cells in the dentate gyrus also express 5-HT2aRs and binding by psilocin may putatively modulate neurogenesis via this receptor. (D) Clinical evidence suggests psilocybin may also be immunomodulatory by moderating cortisol release, HPA axis activity and pro-inflammatory mediator production, notably TNF-α, CRP and IL-6 (Mason et al., 2022). This may directly influence AHN via GR activity. (E) In vitro, Kozlowska et al., 2021 find psilocin may upregulate TREM2 expression, neuronal phagocytosis and modify morphology of primary mouse microglia stimulated with LPS (Kozłowska et al., 2021). Psilocin may putatively bind 5HT2bRs and other 5-HT subtype receptors expressed by microglia perhaps influencing downregulation of pro-inflammatory transcription factors but this has not yet been established. (F) This may also affect pro-inflammatory mediator release by microglia with downstream consequences on other cells in the niche, such as astrocytes, to further influence AHN. (Created with BioRender.com)

Kozłowska et al. (2021) investigated the effect of psilocin on isolated primary CD11b + microglia from mice (Kozłowska et al., 2021, unpublished observation). Psilocin increased TREM2 expression in cells exposed to vehicle but not to LPS. Further, 100uM psilocin, which may be considerably higher than the estimated physiological concentration that produces acute subjective effects in humans (Dahmane et al., 2021), appears to reduce phagocytosis of healthy neurons when microglia are treated with LPS or vehicle. This suggests psilocin may influence microglial phagocytosis and could possibly protect against synapse loss. Pre-treatment with psilocin reduced LPS-induced TLR4 expression and decreased fluorescence of NfKB, a pro-inflammatory gene transcription factor, compared to control (Kozłowska et al., 2021, unpublished observation). Further, they found that psilocin shifted microglia with a ramified morphology after incubation with LPS, to amoeboid morphology. Supporting this connection, bulk RNA sequencing of hippocampus from rats administered psilocybin 0.5–20 mg/kg found gene expression changes related to stress response and neuroplasticity mechanisms; notably upregulation of DUSP1, Iκβ-α and SGK1, and decrease in EGR2 expression (Jefsen, Elfving, Wegener, & Müller, 2021). An in silico model suggested psilacetin, a derivative and prodrug of psilocybin, has the potential to inhibit the IL-6 receptor and modulate cytokine response which may be mediated by 5-HT2aR activation (Khan et al., 2022). Taken together, this suggests psilocybin may directly and indirectly modulate AHN and microglial activity depicted in Fig. 2 and summarised in Table 1.

**Table 1:** Pharmacology of psilocybin and active metabolite psilocin to potentially influence AHN directly or indirectly via activity of HPCs or microglia.

Activity	Clinical	In vivo	In vitro	Direct or indirect relevance to AHN
Modulates adult hippocampal neurogenesis	Psilocybin increases cognitive flexibility in depressed patients (Daws et al., 2022; Doss et al., 2021) which may putatively be mediated by AHN	Psilocybin increases reduced DCX+ and BrdU+ cells in the dentate gyrus in fear conditioned mice associated with fear-extinction. (Du et al., 2023) Psilocybin stimulates extinction of hippocampal-dependent trace fear conditioning in mice that may be associated with changes in neurogenesis indicated by BrdU+ and NeuN+ labelled cells in the dentate gyrus. (Catlow et al., 2013) Putative: Lysergic acid diethylamide (LSD) promotes survival of DGCs in mice which is dependent on BDNF signalling via TrkB (Moliner et al., 2023) Psilocin also promotes neuroplasticity via this mechanism but the activity of psilocybin to stimulate neurogenesis via this mechanism has not yet been established.	There are several receptors present in the hippocampal niche that psilocin may bind to with variable binding affinities, and may influence hippocampal stem cell fate, that has not yet been characterised. This may involve direct or indirect modulation of transporters affecting dopamine signalling via Dopamine Receptor D3 (Ki: 2645), DAT and Dopamine Receptor D2 (Tylš et al., 2023) and serotonergic signalling via 5-HT2C (K_i_: 79-311 nM), 5-HT1A, 5-HT1D (Ki: 36.4), 5-HT7 (Ki: 3.5) and SERT (Ki: 3801) (Erkizia-Santamaría, Alles-Pascual, Horrillo, Meana, & Ortega, 2022b). Psilocin may also bind to Adenosine A2b Receptor (ADORA2B) (Ki: 1894) and Adenosine A2a Receptor (ADORA2A) (Ki: 1379). (Halberstadt & Geyer, 2011)	Direct impact on AHN
Promotes neuroplasticity	5-HT2a receptor occupancy after psilocybin dose associated with hallucinations and antidepressant effects. (Madsen et al., 2019) Psilocybin increases BDNF non-significantly. (Holze et al., 2022)	Psilocybin administration promotes synaptogenesis in the hippocampus and prefrontal cortex, associated with changes in 5HT2aR density, in pigs. (Raval et al., 2021) Psilocybin increases excitatory synapse strength in hippocampus and restoring AMPA/NMDA ratios are associated with reduction in anhedonia-like behaviour in mice. (Hesselgrave et al., 2021) Psilocybin leads to fear extinction in fear-conditioned mice associated with increase in BDNF and mTOR concentration and rescued decrease in hippocampal dendritic complexity. (Du et al., 2023) Psilocybin induces dendritic spine size and density in mice (Shao et al., 2021)	Psilocin activates intracellular 5HT2a receptors and increases dendritic spine density of rat embryonic cortical neurons. (Vargas et al., 2023a) Psilocin is a positive allosteric modulator of BDNF at TrkB by binding to the transmembrane of TrkB dimers to enhance endogenous BDNF, simultaneously increased, signalling leading to dendritogenesis. (Moliner et al., 2023) Putative: psychedelic-induced neuritogenesis, synaptic plasticity, dendritogenesis and spinogenesis may be mediated by mTOR activation (Ly et al., 2018)	Proliferation and differentiation of HPCs are mediated by TrkB activation, mTOR signalling and neurotrophic factors like BDNF. HPCs may express 5-HT2aRs
Immunomodulatory activity	Psilocybin acutely reduces TNFα and increases cortisol followed by reduction in IL-6 and CRP one week after administration in healthy participants. (Mason et al., 2022)	Psilocybin pre-treatment reduced expression of pro-inflammatory COX2, TNFα, IL-1β and IL-6 in mice injected with LPS. (Zanikov et al., 2023)	Psilocin impacts several functions in CD11b + primary mouse microglia stimulated with LPS (Kozłowska et al., 2021): - modified microglial morphology; - reduced TLR4, p65 and CD80 expression and downregulated production of pro-inflammatory gene transcription factor NF-κB; - upregulated TREM2 expression - reduced phagocytosis of healthy neurons Psilocin may bind 5-HT2b (Ki 4.6) and affect CD1+ monocyte-derived dendritic cells to potentially downregulate production of pro-inflammatory cytokines IL-1β, IL-6, TNFα (Chadeayne, Pham, Reid, Golen, & Manke, 2020; Halberstadt & Geyer, 2011; Szabo et al., 2014) Psilocin may bind to Histamine Receptor H1 (Ki 304.6) if expressed by microglia to modulate inflammatory mediator release (Dong et al., 2014)	Indirectly, microglia may be modulated by HPA axis activity and glucocorticoid signalling of HPCs and bi-directional activity of cytokine release on and by microglia. Direct impact on multiple microglial functions and potential downstream modulation of phagocytosis of apoptotic neurons and cytokine release influencing neurogenesis.

## 5 Future directions and clinical considerations

Clinical heterogeneity of both major depression and dementia contributes to diagnostic challenges leading to delayed effective management and reduced quality of life. There are undoubtedly mechanistic commonalities between major depression and dementia where dysregulated adult hippocampal neurogenesis and microglial activity interact at this intersection leading to progressive neuronal loss which may manifest clinically as mood and cognitive impairment.

Broadly, psychedelic compounds have been proposed for dementia management; including for targeting behavioural and psychological symptoms in dementia, treatment of AD, and neuroprotection (George & Hanson, 2019; Kozlowska, Nichols, Wiatr, & Figiel, 2022; Saeger & Olson, 2022; Vann Jones & O'Kelly, 2020; Winkelman, Szabo, & Frecska, 2023). Challenges related to developing disease-modifying therapies for dementia are associated with significant cognitive impairment and therefore atrophy at the point of intervention, which indicates that even if psychedelic compounds target pathological mechanisms of interest, they may not be efficacious in those already diagnosed with dementia. Furthermore, the ethical considerations and potential risks, however rare, of inducing acute hallucinations in a highly vulnerable patient population, particularly in those pre-disposed to psychosis such as in DLB or PDD, which may lead to worsening anxiety, persistent psychosis, or suicidality outweigh any potential benefits. More concerningly, those with reduced capacity may not be able to provide informed consent for psychedelic-assisted therapy. This emphasises the importance of focusing on dementia prevention by developing non-invasive, preclinical biomarkers; highlighting the need for precise stratification to predict who this therapy will be safe and effective for.

It is possible that a subset of depressed individuals, with innate immune system dysregulation and hippocampal-dependent cognitive deficits are at increased risk of developing dementia. Prediction of dementia risk is crucial to preventing cognitive decline and parabiosis assays may be combined with cognitive function tasks such as pattern separation tests which are dentate gyrus-dependent and strongly correlate with hippocampal neurogenesis to aid earlier or preclinical diagnosis of neurodegenerative diseases. Indeed, via investigation of spatial pattern separation deficits in MCI with amnesia, mild AD and cognitively normal age-matched adults, Parizkova et al. (2020) found that spatial pattern separation accuracy was reduced in early AD compared to healthy controls and decreased as disease progressed (Laczó et al., 2021; Parizkova et al., 2020). Spatial pattern separation performance was also strongly associated with hippocampal volume (Gandy et al., 2017). These studies support the idea that prodromal alterations occur in AHN prior to AD onset and may be combined to augment predictive biomarkers. Further, the application of serum-based endogenous factors and their influence on HPCs may enable diagnosis via a less-invasive blood test instead of cerebrospinal fluid collection (De Lucia et al., 2022).

Although there are significant challenges associated with the therapeutic development of psychedelics, psilocybin may be advantageous over currently available antidepressants due to rapid onset of action, reduced dosing frequency requirements and potentially fewer undesirable effects which may contribute to medication compliance. In this regard, psilocybin demonstrates significant clinical development potential for dementia prevention only and may directly and indirectly influence mechanisms mediated by AHN and microglia at the nexus of neurodegenerative diseases and major depression. Further, the development of translatable preclinical models to establish how psilocybin exerts these effects relative to clinical response, including effects on cognitive function, as well as the appropriate dose and frequency of administration, is needed. Whether psilocybin effectively modulates pathological processes to alter the trajectory of neurodegeneration and delay cognitive decline in those at risk is yet to be determined, but the current evidence demonstrates promise.

## 6 Conclusion

Major depression and dementia are both associated with significant morbidity and have rippling emotional and socioeconomic consequences. Notably, dementia may be preventable and evidence suggests targeting major depression as a modifiable risk factor may delay cognitive decline, but timing is crucial. Unravelling the pathological underpinnings related to MDD may aid in identifying new disease biomarkers and drug targets. Hippocampal volume loss, altered adult hippocampal neurogenesis, raised pro-inflammatory mediators and dysregulated microglia are associated with both conditions. Drugs that improve mood may also benefit cognitive function by influencing the trajectory of neurodegeneration. However, there is limited and conflicting evidence to suggest currently available antidepressants adequately target the intersecting processes associated with pathological AHN and microglial activity.

Psilocybin is capable of improving depressive symptoms, promoting cognitive flexibility and may exhibit multi-modal mechanisms of action, which may influence AHN-specific signalling pathways in MDD. Psilocybin directly alters adult hippocampal neurogenesis in animal models via increasing BDNF, which may be mediated by initial spikes in cortisol, whereby neuronal TrkB receptor activation leads to multiple intracellular signalling pathways to increase neuritogenesis, spinogenesis and neuron survival influencing synaptic plasticity. Indirectly, through cortisol and HPA axis modulation, psilocybin may influence cytokine production and influence 5-HT receptors on microglia which directly interact with DG precursor cells in the hippocampal niche. The downstream effects of this may also affect phagocytosis of apoptotic progenitor cells and synapses, perhaps mediated by pro- and anti-inflammatory cytokine and BDNF production in the neurogenic niche.

The current evidence implies that psilocybin may potentially be neuroprotective and promote stress resilience. This is promising but further translational, mechanistic research expanding on the role of psilocybin, and its active metabolites, on microglial functions and inflammatory mediators related to AHN is needed; particularly in the context of hippocampal-dependent cognitive function in MDD and neurodegenerative diseases.

## CRediT authorship contribution statement

**Zarah R. Haniff:** Writing – review & editing, Writing – original draft, Visualization, Conceptualization. **Mariia Bocharova:** Writing – review & editing, Writing – original draft. **Tim Mantingh:** Writing – review & editing. **James J. Rucker:** Writing – review & editing. **Latha Velayudhan:** Writing – review & editing. **David M. Taylor:** Writing – review & editing. **Allan H. Young:** Writing – review & editing, Conceptualization. **Dag Aarsland:** Writing – review & editing, Conceptualization. **Anthony C. Vernon:** Writing – review & editing, Conceptualization. **Sandrine Thuret:** Writing – review & editing, Conceptualization.

## Declaration of competing interest

DA, MB, TM, ST, LV and ACV declare no conflict of interest relevant to the content of this manuscript.

ZRH: Investigational material received from COMPASS Pathways.

DMT: Shareholder of Myogenes and 428 Pharma.

JJR: Paid advisory boards for Clerkenwell Health (Past), Beckley PsyTech (Past), Delica Therapeutics (Past). Paid articles for Janssen. Assistance for attendance at conferences from Compass Pathways (past) and Janssen. Grant funding (received and managed by King's College London) from Compass Pathfinder, Beckley PsyTech, Multidisciplinary Association for Psychedelic Studies, National Institute for Health Research, Wellcome Trust, Biomedical Research Centre at the South London and Maudsley NHS Foundation Trust. No shareholdings in pharmaceutical companies. No shareholdings in companies developing psychedelics.

AHY: Employed by King's College London; Honorary Consultant South London and Maudsley NHS Foundation Trust (NHS UK). Editor of Journal of Psychopharmacology and Deputy Editor, BJPsych Open. Paid lectures and advisory boards for the following companies with drugs used in affective and related disorders: FlowNeuroscience, Novartis, Roche, Janssen, Takeda, Noema pharma, Compass, Astrazenaca, Boehringer Ingelheim, Eli Lilly, LivaNova, Lundbeck, Sunovion, Servier, Livanova, Janssen, Allegan, Bionomics, Sumitomo Dainippon Pharma, Sage, Novartis, Neurocentrx. Principal Investigator in the Restore-Life VNS registry study funded by LivaNova. Principal Investigator on ESKETINTRD3004: “An Open-label, Long-term, Safety and Efficacy Study of Intranasal Esketamine in Treatment-resistant Depression.” Principal Investigator on “The Effects of Psilocybin on Cognitive Function in Healthy Participants”. Principal Investigator on “The Safety and Efficacy of Psilocybin in Participants with Treatment-Resistant Depression (P-TRD)”. Principal Investigator on “A Double-Blind, Randomized, Parallel-Group Study with Quetiapine Extended Release as Comparator to Evaluate the Efficacy and Safety of Seltorexant 20 mg as Adjunctive Therapy to Antidepressants in Adult and Elderly Patients with Major Depressive Disorder with Insomnia Symptoms Who Have Responded Inadequately to Antidepressant Therapy.” (Janssen). Principal Investigator on “ An Open-label, Long-term, Safety and Efficacy Study of Aticaprant as Adjunctive Therapy in Adult and Elderly Participants with Major Depressive Disorder (MDD).” (Janssen). Principal Investigator on “A Randomized, Double-blind, Multicentre, Parallel-group, Placebo-controlled Study to Evaluate the Efficacy, Safety, and Tolerability of Aticaprant 10 mg as Adjunctive Therapy in Adult Participants with Major Depressive Disorder (MDD) with Moderate-to-severe Anhedonia and Inadequate Response to Current Antidepressant Therapy”. Principal Investigator on “ A Study of Disease Characteristics and Real-life Standard of Care Effectiveness in Patients with Major Depressive Disorder (MDD) With Anhedonia and Inadequate Response to Current Antidepressant Therapy Including an SSRI or SNR.” (Janssen). UK Chief Investigator for Compass; COMP006 & COMP007 studies. UK Chief Investigator for Novartis MDD study MIJ821A12201. Grant funding (past and present): NIMH (USA); CIHR (Canada); NARSAD (USA); Stanley Medical Research Institute (USA); MRC (UK); Wellcome Trust (UK); Royal College of Physicians (Edin); BMA (UK); UBC-VGH Foundation (Canada); WEDC (Canada); CCS Depression Research Fund (Canada); MSFHR (Canada); NIHR (UK). Janssen (UK) EU Horizon 2020. No shareholdings in pharmaceutical companies.
